# Dietary energy sources and levels shift the multi-kingdom microbiota and functions in the rumen of lactating dairy cows

**DOI:** 10.1186/s40104-020-00461-2

**Published:** 2020-06-22

**Authors:** Tansol Park, Lu Ma, Ying Ma, Xiaoqiao Zhou, Dengpan Bu, Zhongtang Yu

**Affiliations:** 1 Department of Animal Sciences, The Ohio State University, Columbus, OH USA; 2 The State Key Laboratory of Animal Nutrition, Institute of Animal Science, Chinese Academy of Agricultural Sciences, Beijing, P. R. China; 3 CAAS-ICRAF Joint Lab on Agroforestry and Sustainable Animal Husbandry, Beijing, P. R. China

**Keywords:** Corn processing method, Dietary energy level, Functional profiles, Multi-kingdom, Rumen microbiota

## Abstract

**Background:**

Dietary energy source and level in lactation diets can profoundly affect milk yield and composition. Such dietary effects on lactation performance are underpinned by alteration of the rumen microbiota, of which bacteria, archaea, fungi, and protozoa may vary differently. However, few studies have examined all the four groups of rumen microbes. This study investigated the effect of both the level and source of dietary energy on rumen bacteria, archaea, fungi, and protozoa in the rumen of lactating dairy cows. A 2 × 2 factorial design resulted in four dietary treatments: low and high dietary energy levels (LE: 1.52–1.53; and HE: 1.71–1.72 Mcal/kg dry matter) and two dietary energy sources (GC: finely ground corn; and SFC: steam-flaked corn). We used a replicated 4 × 4 Latin square design using eight primiparous Chinese Holstein cows with each period lasting for 21 d. The rumen microbiota was analyzed using metataxonomics based on kingdom-specific phylogenetic markers [16S rRNA gene for bacteria and archaea, 18S rRNA gene for protozoa, and internally transcribed spacer 1 (ITS1) for fungi] followed with subsequent functional prediction using PICRUSt2.

**Results:**

The GC resulted in a higher prokaryotic (bacterial and archaeal) species richness and Faith’s phylogenetic diversity than SFC. For the eukaryotic (fungi and protozoa) microbiota, the LE diets led to significantly higher values of the above measurements than the HE diets. Among the major classified taxa, 23 genera across all the kingdoms differed in relative abundance between the two dietary energy levels, while only six genera (none being protozoal) were differentially abundant between the two energy sources. Based on prokaryotic amplicon sequence variants (ASVs) from all the samples, overall functional profiles predicted using PICRUSt2 differed significantly between LE and HE but not between the two energy sources. FishTaco analysis identified *Ruminococcus* and *Coprococcus* as the taxa potentially contributing to the enriched KEGG pathways for biosynthesis of amino acids and to the metabolisms of pyruvate, glycerophospholipid, and nicotinate and nicotinamide in the rumen of HE-fed cows. The co-occurrence networks were also affected by the dietary treatments, especially the LE and GC diets, resulting in distinct co-occurrence networks. Several microbial genera appeared to be strongly correlated with one or more lactation traits.

**Conclusions:**

Dietary energy level affected the overall rumen multi-kingdom microbiota while little difference was noted between ground corn and steam-flaked corn. Some genera were also affected differently by the four dietary treatments, including genera that had been shown to be correlated with lactation performance or feed efficiency. The co-occurrence patterns among the genera exclusively found for each dietary treatment may suggest possible metabolic interactions specifically affected by the dietary treatment. Some of the major taxa were positively correlated to milk properties and may potentially serve as biomarkers of one or more lactation traits.

## Introduction

The rumen microbiota consists of bacteria, archaea, fungi, protozoa, and viruses, and its diversity and functional versatility enable robust utilization of various feed ingredients including recalcitrant plant wall materials, producing assimilable energy and nutrients required by ruminants [1]. Dietary interventions such as supplementation with feed additives and alterations of feed composition and digestibility are frequently used to optimize productivity (average daily gain and milk yield), improve product quality, and minimize animal health issues [2]. Indubitably, the effects and efficacy of these dietary interventions are made possible because of the rumen microbiota. Indeed, many of the production phenotypes of ruminant animals are associated with the rumen microbiota and its functionality [3–5]. However, few studies have analyzed bacteria, archaea, fungi, and protozoa together, missing the opportunity to examine how dietary interventions affect the interactions among these groups of prokaryotes and eukaryotes, which are important to the understanding of the microbial underpinning of treatment effects [6].

In dairy cows, the forage-to-concentrate ratio [7], source of forage [8], dietary energy source and level [9], and synchronization of dietary energy and nitrogen [10] are the major nutritional factors that determine milk yield and quality and are thus areas of extensive research. High-quality forage (e.g., corn silage and alfalfa hay) and energy-dense feed (e.g., corn) support high lactation performance, but in most countries outside of North America and Europe, these feed ingredients are limited in quantity and expensive, while low-quality forages, such as corn stover (CS) and rice straw, are abundant. Low-quality forages often decrease the metabolizable energy (ME) content of the diets because of their poor palatability, low digestibility, and low content of crude protein and non-fiber readily fermentable carbohydrates [8, 11, 12]. As a result, ruminal microbial protein (MP) synthesis and dietary energy supply to the host animals are inadequate to support high lactation performance [8, 12, 13]. Corn is the most energy-dense feed fed to dairy cows, and steam-flaking has been one of the most commonly used corn processing technologies to increase the digestibility of intact corn grains. Inclusion of steam-flaked corn (SFC) results in improved MP synthesis and lactation performance in lactating dairy cows [14, 15]. Conceivably, the rumen microbiota will respond to the changes in source and level of forage and ME in the diet.

In a previous study investigating how dietary energy source and level affect feed digestibility, MP synthesis, and lactation performance of dairy cows, we found that total-tract apparent digestibility, milk yield, milk protein content and yield, and milk lactose yield all increased with increase in dietary energy, and when SFC replaced ground corn, milk yield, milk protein yield, and MP yield also increased [9]. We hypothesized that the observed differences in lactation performance might have resulted from the alteration of the rumen microbiota responding to the different energy sources and levels. The objective of this study was to examine how and to what extent the different sources and levels of dietary energy had affected the rumen microbiota and their interactions among themselves and with lactation performance phenotypes. To achieve a holistic view, bacteria, archaea, fungi, and protozoa were analyzed simultaneously using metataxonomics.

## Methods

### Animals and diets

The feeding experiment has been reported in a previous article [9]. Briefly, animal usage was approved by the Animal Care Advisory Committee of the Chinese Academy of Agricultural Sciences. A 2 × 2 factorial design resulted in four dietary treatments: low and high energy levels (LE: 1.52–1.53; and HE: 1.71–1.72 Mcal/kg of dry matter) and two energy sources (GC: finely ground corn; and SFC: steam-flaked corn). The feed composition of the four dietary treatments was presented in Table S1. We used eight primiparous Chinese Holstein cows in a replicated 4 × 4 Latin square design with each period lasting for 21 d. Four of the cows were each fitted with a rumen cannula and assigned to one of the four treatments. The animals were fed twice daily (06:30 and 16:30 h) for ad libitum intake, allowing for 5% orts, with free access to clean drinking water. Cows were milked thrice daily, at 07:00, 13:30, and 20:30 h. The rumen fluid samples were collected 3 h after the morning feeding on d 18 using an oral stomach tube and then stored at − 80 °C until metagenomic DNA extraction.

### DNA extraction and metataxonomic analysis of multi-kingdom rumen microbiota

Metagenomic DNA from each rumen sample was extracted using the RBB + C method [16]. The quality and quantity of the extracted metagenomic DNA were evaluated using a NanoDrop ND-2000 Spectrophotometer (Thermo Scientific, NanoDrop Technologies, Wilmington, DE, USA) followed by agarose gel (1%, wt/vol) electrophoresis. The rumen microbiota was analyzed using metataxonomics based on kingdom-specific phylogenetic markers (16S rRNA gene for bacteria and archaea, 18S rRNA gene for protozoa, and internally transcribed spacer 1 (ITS1) for fungi). Briefly, one amplicon library for each rumen sample was prepared for each target microbial group by PCR amplification using the respective primer sets (Table S2). All the amplicon libraries had a unique barcode for multiplexing. The amplicon libraries for prokaryotes, protozoa, and fungi for each sample were pooled at 9: 0.5: 0.5 ratio first, resulting in one pooled amplicon library for each sample. Then, pooled amplicon libraries for all the samples were pooled again at equal ratio and sequenced using the 2 × 300 paired-end protocol on the Illumina MiSeq platform. Raw sequencing reads have been deposited in the NCBI Sequence Read Archive (SRA) under BioProject PRJNA523854.

The amplicon sequences were analyzed using QIIME2 with built-in commands and plugins (version 2019.4) [17]. Briefly, adapter and primer sequences were removed using Cutadapt [18] followed by quality filtering (Q-score ≥ 25), denoising, merging, and chimeric sequence removal as done previously [19] using the DADA2 plugin [20]. Specifically for fungal sequences, ITSxpress [21] was used to delineate the ITS1 region followed by dereplication and chimera removal using q2-dada2’s denoise-single method [20]. Amplicon sequence variants (ASVs) were clustered at 99% similarity. Taxonomic classifiers for each multi-kingdom were manually trained using the Naïve Bayes classifier [22] with the Greengenes 16S reference database (13_8 version) for bacteria and archaea, the UNITE ITS reference sequences (10.10.2017 database) for fungi, and the Silva 18S reference database (NR 132 version) for protozoa. Major phyla and genera each representing ≥0.1% of total sequences in at least one of the dietary treatments were discussed in this report.

Alpha-diversity measurements including richness (ASV, genera, and species), evenness, Faith’s phylogenetic diversity, Shannon’s diversity index, and Simpson’s index were assessed based on the rarefied ASV BIOM tables. The overall microbiotas shaped by the different dietary treatments were compared using principal coordinates analysis (PCoA) based on the weighted UniFrac distances. With combined multi-kingdom compositional data at the genus level (major known genera only), Sparse Co-occurrence Network Investigation for Compositional data (SCNIC) was used to examine the correlation based on the co-occurrence and mutual-exclusion networks for each dietary treatment by computing Spearman correlation coefficients (https://github.com/shafferm/SCNIC). Only significant interactions (*P* < 0.05) were further analyzed using Co-expression Differential Network Analysis (CoDiNA) [23] to identify specific microbial networks at each dietary treatment.

### Functional prediction and identification of taxonomic drivers

Metabolic functions were predicted from 16S- and ITS1-based ASVs using Phylogenetic Investigation of Communities by Reconstruction of Unobserved States 2 (PICRUSt2) [24]. The identified functional features were determined using six different reference databases (Table 2). Overall functional dissimilarities among different dietary treatments were examined based on the relative abundance of predicted enzymes assigned to the Enzyme Nomenclature database (ENZYME, https://enzyme.expasy.org/) for both prokaryotic- and fungal-microbiota and then visualized using principal components analysis (PCA) based on Bray-Curtis dissimilarities. Functional Shifts’ Taxonomic Contributors (FishTaco) [25] was used to define both the differentially abundant KEGG pathways between two different dietary treatments and the corresponding taxonomic drivers of each detected pathway. Functional shifts of defined KEGG pathways attributable to major taxonomic drivers at the genus level of rumen prokaryotes were visualized using the FishTacoPlot R package (https://github.com/borenstein-lab/fishtaco-plot).

### Calculations and statistical analysis

Alpha diversity measurements of each microbial community (bacterial and archaeal, fungal, or protozoal) and the counts of PICRUSt2-predicted functional features were statistically analyzed using the GLIMMIX procedure of SAS 9.4 (SAS Institute Inc., Cary, NC, USA):

$$ {\mathrm{Y}}_{\mathrm{i}\mathrm{j}\mathrm{k}}=\mathrm{u}+{\mathrm{T}}_{\mathrm{i}}+{\mathrm{H}}_{\mathrm{j}}+{\mathrm{T}}_{\mathrm{i}}{\mathrm{H}}_{\mathrm{j}}+{\mathrm{e}}_{\mathrm{i}\mathrm{j}}, $$

where *μ* is the mean of alpha diversity measurements or the number of functional features, *T*_*i*_ is the fixed effect of the dietary energy level (*i* = 1, 2), *H*_*j*_ is the fixed effect of energy source (*j* = 1, 2), and *e*_*ij*_ is the residual error. *T*_*i*_*H*_*j*_ is the fixed effect of the interaction between ith dietary energy level and the *j*^th^ energy source. Permutational multivariate analysis of variance (PERMANOVA) was used to further analyze the PCoA and PCA results to assess if each microbial community significantly differed between the two dietary energy levels or between the two energy sources using PAST3 [26] with 9999 random permutations. Differentially abundant microbial taxa between different dietary energy levels and between the two energy sources were identified using linear discriminant analysis (LDA) effect size (LEfSe) [27] with a logarithmic LDA score 2 as the cutoff. Pearson correlation coefficients between milk yield and milk components reported in the previous animal study [9] and the relative abundance of major known microbial genera determined in the present study were computed using the PROC CORR procedure in SAS and visualized using the R (v.3.5.0) package “corrplot”. Statistical significance was declared at *P* ≤ 0.05 and tendency at 0.05 < *P* ≤ 0.10.

## Results

### Dietary energy level affects alpha and beta diversity and co-occurrence interactions

**Fig. 1:**
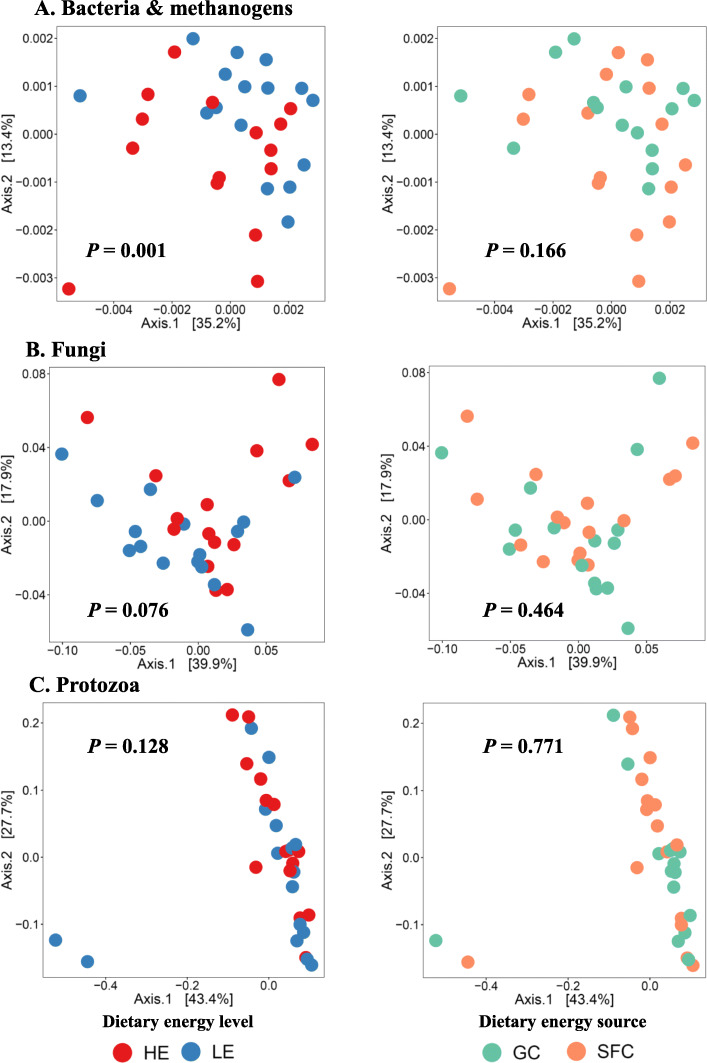
Plots of principal coordinates analysis (PCoA) of the rumen microbiota based on weighted UniFrac distance matrices. HE, high energy; LE, low energy; GC, ground corn; SFC, steam-flaked corn. *P*-values were based on PERMANOVA

After quality filtering and rarefaction, 36348, 1213, and 4981 sequences per sample were obtained for bacteria and archaea, fungi, and protozoa, respectively, which resulted in greater than 96.8% of Good’s coverage for all the samples (data not shown). In the prokaryotic microbiota, HE significantly decreased the number of observed species and genera, while SFC corresponded to decreased numbers of observed ASVs and a lower Faith’s phylogenetic diversity than GC (Table 1). The high dietary energy level significantly decreased the number of ASVs, species, genera, and Shannon diversity index while increasing Faith’s phylogenetic diversity of the fungal microbiota. As for the protozoal microbiota, HE decreased taxa richness (ASVs, species, and genera) and Faith’s phylogenetic diversity. The two energy sources (GC vs. SFC) did not result in significant changes in any of the alpha diversity measurements of the fungal or the protozoal microbiotas. The PCoA based on the weighted UniFrac distance showed that the dietary energy level significantly affected the prokaryotic microbiota and tended to affect the fungal microbiota, but not the protozoa microbiota (Fig. 1). The two energy sources did not significantly alter the overall prokaryotic or eukaryotic microbiotas.

**Table 1:** Alpha diversity measurements of the ruminal microbiota

Measurements	LE		HE		SEM	*P*-values			
	GC	SFC	GC	SFC		NE	ES	Period	NE × ES
Bacteria and archaea									
Observed ASVs	1050	808	973	752	45.211	0.3456	**0.0116**	0.1646	0.7936
Observed genera	110^A^	100^AB^	100^AB^	91^B^	2.738	**0.0368**	*0.0756*	0.3117	0.8548
Observed species	126^A^	115^AB^	113^AB^	104^B^	3.127	**0.0360**	0.1006	0.3115	0.7053
Shannon	8.27	8.24	8.22	8.00	0.063	0.2532	0.3962	0.8501	0.5160
Simpson	0.99	0.99	0.99	0.99	0.001	0.7580	0.5245	**<.0001**	0.3247
Faith’s PD	57.66	50.46	54.08	48.65	1.429	0.2187	**0.0285**	0.1718	0.5956
Evenness	0.83	0.86	0.83	0.84	0.006	0.4262	0.1314	0.5571	0.4853
Fungi									
Observed ASVs	97^AB^	101^A^	71^AB^	68^B^	4.511	**0.0008**	0.9189	0.6974	0.7098
Observed genera	50	52	41	40	1.947	**0.0107**	0.8837	0.7432	0.7498
Observed species	63	64	47	49	2.931	**0.0106**	0.7737	0.8287	0.9804
Shannon	5.33	5.46	4.91	5.03	0.075	**0.0052**	0.4194	0.3448	0.8850
Simpson	0.95	0.96	0.94	0.95	0.003	*0.0794*	0.1340	0.1374	0.8218
Faith’s PD	25.75	26.58	20.36	22.03	0.989	**0.0128**	0.4742	0.6513	0.7740
Evenness	0.81	0.82	0.81	0.83	0.007	0.7033	0.2685	**0.0047**	0.7557
Protozoa									
Observed ASVs	73^A^	69^AB^	61^AB^	61^B^	2.275	**0.0051**	0.4157	0.6152	0.5865
Observed genera	5	5	4	4	0.153	**0.0131**	0.6678	0.4967	0.3796
Observed species	10^A^	9^AB^	8^B^	9^AB^	0.243	**0.0046**	0.7940	0.5114	0.2616
Shannon	4.79	4.62	4.61	4.54	0.116	0.4194	0.4437	0.6601	0.7803
Simpson	0.93	0.91	0.94	0.93	0.012	0.4435	0.5476	0.4481	0.5636
Faith’s PD	0.87	0.82	0.72	0.76	0.024	**0.0133**	0.9700	0.7342	0.2230
Evenness	0.77	0.76	0.78	0.77	0.015	0.7145	0.5960	0.5357	0.9009

*LE* Low energy, *HE* High energy, *GC* Ground corn, *SFC* Steam-flaked corn, *NE* Effect of dietary energy level, *ES* Effect of energy sources, *NE × ES* Interaction between NE and ES

*ASVs* Amplicon sequence variants

*Faith’s PD* Faith’s phylogenetic diversity

Different superscripts in upper case within a row indicate significant difference (*P* ≤ 0.05). The *P*-values ≤0.05 are bolded, while those > 0.05 but ≤0.1 are italicized

**Fig. 2:**
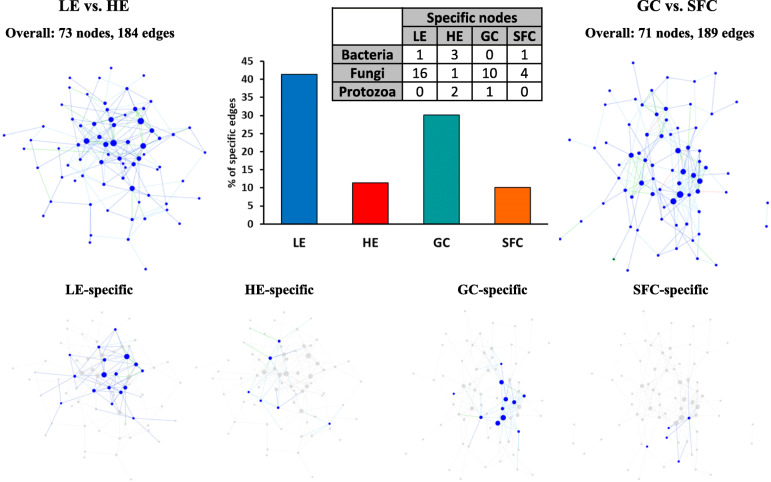
Co-occurrence network based on the significant correlations (*P* < 0.05) based on relative abundance of major genera (each having a relative abundance ≥0.1% in at least one of the dietary treatments) of rumen microbiota. The microbial taxa nodes exclusively found in each dietary treatment were also presented. The size of the nodes represents the sum of the weights of the interactions connected. HE, high energy; LE, low energy; GC, ground corn; SFC, steam-flaked corn. LE-specific nodes: one bacterial genus (*Succinivibrio*) and 16 fungal genera (*Pyrenochaetopsis*, *Debaryomyces*, *Epicoccum*, *Orpinomyces*, *Gibberella*, *Bullera*, *Hannaella*, *Alternaria*, *Papiliotrema*, *Dioszegia*, *Penicillium*, *Cladosporium*, *Dipodascus*, *Saitozyma*, *Acremonium*, and *Sporobolomyces*); HE-specific nodes: three bacterial genera (*Prevotella*, RFN20, and BF311), one fungal genus (*Meyerozyma*), and two protozoal genera (*Dasytricha* and *Entodinium*); GC-specific nodes: 10 fungal genera (*Erythrobasidium*, *Sarocladium*, *Papiliotrema*, *Vishniacozyma*, *Acremonium*, *Sporobolomyces*, *Phaeosphaeria*, *Gibberella*, *Saitozyma*, and *Cladosporium*) and one protozoal genus (*Entodinium*); SFC-specific nodes: one bacterial genus (*Ruminobacter*) and four fungal genera (*Hanseniaspora*, *Cyberlindnera*, *Kurtzmanomyces*, and *Alternaria*)

Compositional data-based network analysis identified 73 and 71 microbial nodes with 184 and 189 significant interactions among the identified genera (*P* < 0.05) for the two energy levels and the two energy sources, respectively (Fig. 2). When the significant co-occurrence and mutual exclusion interactions were compared between the two dietary energy levels or the two energy sources, more interactions were specifically assigned to LE or GC. Particularly, fungal populations were mainly responsible for those interactions.

### The two dietary energy levels, but not the two energy sources, affect some taxa of the rumen microbiota

Irrespective of the dietary treatments, Bacteroidetes is the most predominant phylum accounting for at least 65% of the total prokaryotic microbiota, while Firmicutes and Proteobacteria came in second and third, respectively (Fig. S1). At the genus level, *Prevotella* represented about half of the entire prokaryotic community, and roughly 70% of the total prokaryotic sequences could be assigned to 24 major classified genera (each representing ≥0.1% of total sequences in at least one of the dietary treatments). Three major fungal phyla, Ascomycota, Basidiomycota, and Neocallimastigomycota, represented over 94% in all the dietary treatments. Two genera within Neocallimastigomycota (i.e., *Orpinomyces* and *Piromyces*) and *Candida* within Ascomycota were the most predominant fungal genera regardless of dietary treatments. Only five protozoal genera (i.e., *Dasytricha*, *Entodinium*, *Isotricha*, *Ophryoscolex*, and *Polyplastron*) were detected among the dairy cows. Overall, *Entodinium* was the most predominant protozoal genus, but its relative abundances varied from 52% to 73% depending on the dietary treatments.

**Fig. 3:**
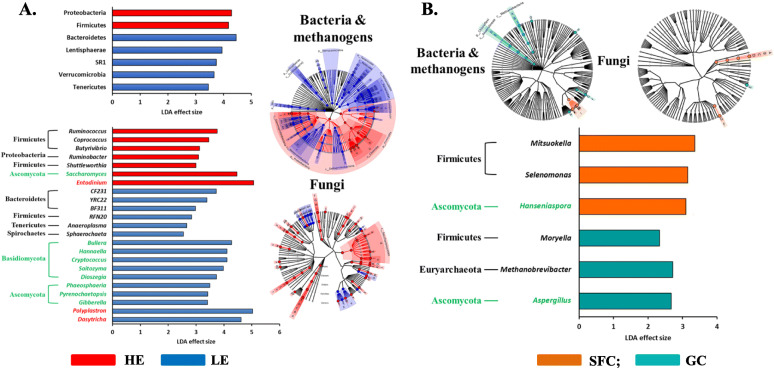
Differentially abundant microbial genera as affected by energy levels (**a**) and energy sources (**b**), which were detected using LEfSe with an LDA effect size ≥2. Only major classified taxa (each representing ≥0.1% in at least one of the dietary treatments) were visualized with additional taxonomic lineages of prokaryotes and fungi embedded in a respective cladogram. HE, high energy; LE, low energy; GC, ground corn; SFC, steam-flaked corn

The differentially abundant genera of the rumen microbiota between the different dietary energy levels and between the different dietary sources were identified using LEfSe (Fig. 3). At the phylum level of bacteria, the relative abundance of Bacteroidetes, Lentisphaerae, SR1, Verrucomicrobia, and Tenericutes were enriched in the cows fed the LE diet, whereas that of Proteobacteria and Firmicutes were enriched in the cows fed the HE diets. The two dietary energy sources did not result in the enrichment of any of the major bacterial phyla. Three candidate genera within Bacteroidetes (i.e., CF231, YRC22, and BF311), one candidate genus within Firmicutes (i.e., RFN20), *Anaeroplasma*, and *Sphaerochaeta* were differentially abundant in the cows fed the LE diet. All the five differentially abundant bacterial genera identified in the cows fed the HE diets belonged to either Firmicutes or Proteobacteria. No fungal phyla were found to be differentially abundant, but eight major fungal genera were enriched in the cows fed the LE diet, while *Saccharomyces* was enriched in the cows fed the HE diets. *Aspergillus* and *Hanseniaspora* expanded relative abundance in the cows fed GC and SFC, respectively. The LE diet increased the relative abundance of *Dasytricha* and *Polyplastron*, while the HE diets increased the predominance of *Entodinium*. The two energy sources did not cause a significant difference in the relative abundance of any of the protozoal genera.

### The two dietary energy levels, but not the two energy sources, affect some predicted functions of rumen prokaryotes

**Fig. 4:**
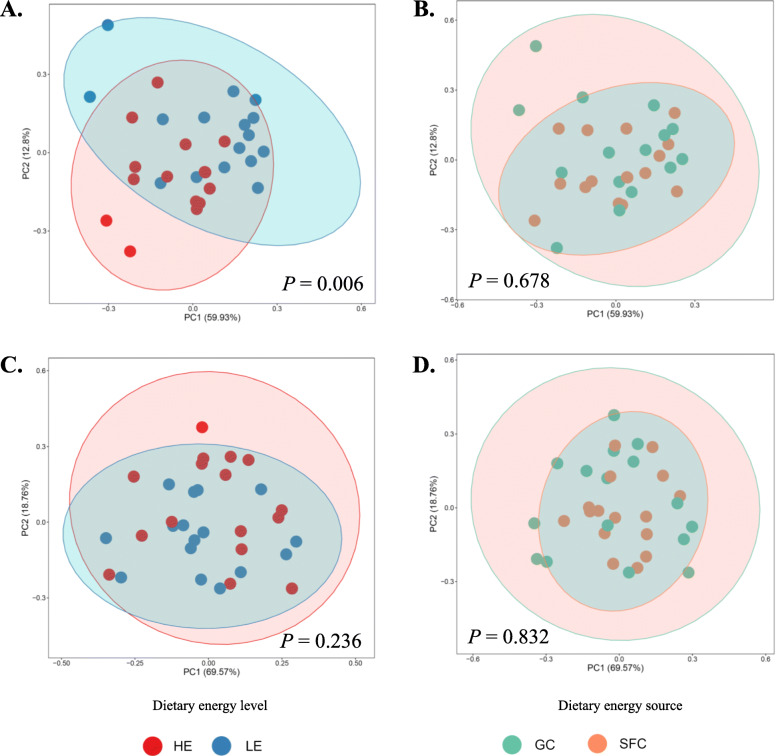
PCA plots comparing the overall functional features of the rumen prokaryotic (**a** and **b**) and fungal (**c** and **d**) microbiota between LE and HE (**a** and **c**) or GC and SFC (**b** and **d**). Functional features were predicted using PICRUSt2 and the ExPASy ENZYME database. HE, high energy; LE, low energy; GC, ground corn; SFC, steam-flaked corn. *P*-values were based on PERMANOVA

**Fig. 5:**
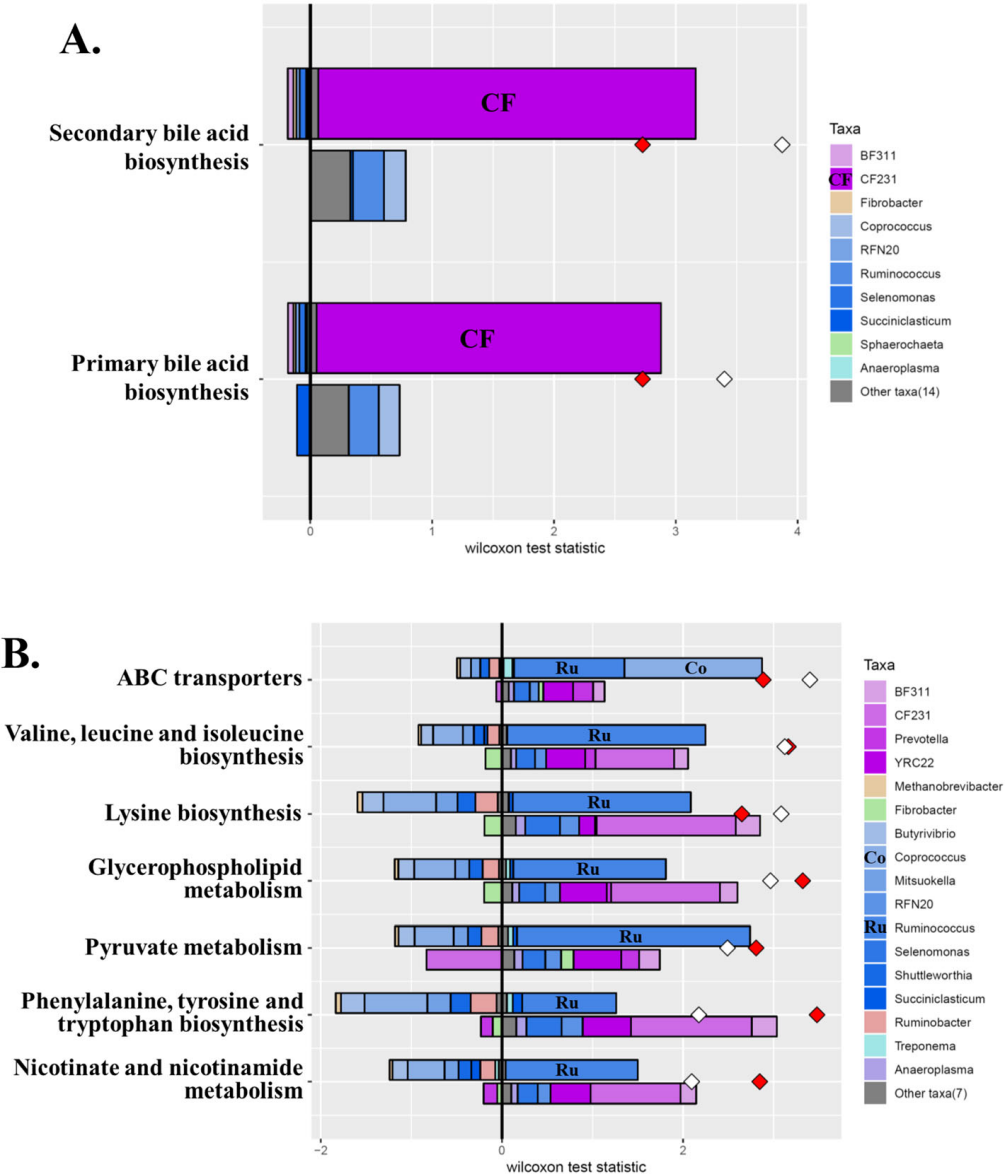
Taxonomic drivers of differentially abundant KEGG pathways detected with FishTaco (only those with a z-score ≥ 2 of Wilcoxon rank-sum test are shown). Only the major prokaryotic genera (each having a relative abundance ≥0.1% in at least one of the dietary treatments) were included in the PICRUSt2 and FishTaco analyses. **a** taxonomic drivers and KEGG pathways exclusively found in the cows fed LE diets; **b** taxonomic drivers and KEGG pathways exclusively found in the cows fed LE diets. The two energy sources did not result in significant difference in the predicted KEGG pathways

Various functional features were predicted from the 16S rRNA gene sequences and ITS1 sequences using six different reference databases (Table 2). The dietary energy levels significantly affected the numbers of functional features, with an overall increase at least numerically by the LE diets irrespective of the energy sources. On the other hand, the two different energy sourced did not result in significant differences in any of the predicted functional features irrespective of the databases used. The overall distributions of prokaryotic and fungal enzymes predicted based on EC numbers were visualized in PCA plots (Fig. 4). Based on PERMANOVA analysis, the two dietary energy levels resulted in significantly different (*P* = 0.006) distributions of prokaryotic enzymes (based on EC numbers), while the two energy sources did not cause any difference (*P* = 0.678). The predicted categories of fungal enzymes did not differ between the two dietary energy levels or between the two energy sources. The relative abundances of the predicted enzymes were only different between the two dietary energy levels but not between the two dietary energy sources (Table 3). The two energy levels resulted in different abundance in 16 types of prokaryotic enzymes (based on EC numbers; including four EC 1, oxidoreductases; five EC 2, transferases; five EC 3, hydrolases; and two EC 5, isomerases) and 13 types of fungal enzymes (including one EC 1, oxidoreductases; three EC 2, transferases; six EC 3, hydrolases; two EC 2, lyases; and one EC 5, isomerases). Further analysis using FishTaco identified two KEGG pathways (i.e., ko00121, secondary bile acid biosynthesis; ko00120, primary bile acid biosynthesis) that were enriched by the LE diet, and the functional shifts were mainly associated with a candidate genus CF231 (Fig. 5a). In response to the HE diet, seven KEGG pathways (i.e., ko02010, ABC transporters; ko00290, valine, leucine and isoleucine biosynthesis; ko00300, lysine biosynthesis; ko00564, glycerophospholipid metabolism; ko00620, pyruvate metabolism; ko00400, phenylalanine, tyrosine and tryptophan biosynthesis; ko00760, nicotinate and nicotinamide metabolism) were enriched, and the enrichment was primarily contributed by *Coprococcus* and *Ruminococcus* (Fig. 5b).

**Table 2:** Predicted KEGG hierarchies (orthologs, modules, and pathways), Protein families (Pfam), Clusters of Orthologous Genes (COG), Enzyme Classification numbers (EC numbers), and MetaCyc pathways counts in each treatment

Measurements	LE		HE		SEM	*P*-values			
	GC	SFC	GC	SFC		NE	ES	Period	NE × ES
KEGG orthologs	5731	5563	5351	5290	67	**0.0126**	0.3778	0.5038	0.6221
KEGG modules	282	278	280	276	1	0.5080	0.1011	0.5542	0.9846
KEGG pathways	145^A^	143^AB^	140^B^	141^AB^	1	**0.0059**	0.8504	0.3523	0.2589
PFAM	6757^a^	6595^ab^	6384^ab^	6329^b^	68	**0.0120**	0.3980	0.3932	0.6092
COG	4181^a^	4111^ab^	4064^ab^	4045^b^	22	**0.0283**	0.2666	0.7715	0.5084
EC numbers	1816^A^	1757^AB^	1715^AB^	1697^B^	16	**0.0096**	0.2191	0.3870	0.4541
Fungal EC numbers	989^A^	984^AB^	972^AB^	966^B^	3	**0.0016**	0.3540	0.3209	0.9354
MetaCyc pathways	369^A^	361^AB^	343^B^	345^AB^	4	**0.0035**	0.6464	0.5974	0.4276

*LE* Low energy, *HE* High energy, *GC* ground corn, *SFC* Steam-flaked corn, *NE* Effect of dietary energy level, *ES* effect of energy sources, *NE × ES* Interaction between NE and ES

Different superscripts within a row indicate significant difference (*P* ≤ 0.05), while different superscripts in lower case indicate tendency (*P* ≤ 0.1). The *P*-values ≤0.05 are bolded

**Table 3:** Differentially abundant KEGG pathways affected by energy levels which were detected using LEfSe with an LDA effect size ≥2.0

Item	EC numbers	Relative abundance, %		SEM	Class	LDA score	*P*-value	Description
		LE	HE					
Prokaryotic	EC 1.2.7.11	0.506	0.467	0.007	LE	2.312	0.009	2-oxoacid oxidoreductase (ferredoxin)
	EC 1.2.7.3	0.762	0.704	0.011	LE	2.483	0.013	2-oxoglutarate synthase
	EC 1.3.5.1	0.271	0.248	0.004	LE	2.075	0.001	Succinate dehydrogenase (quinone)
	EC 1.6.5.3	1.333	1.279	0.010	LE	2.424	0.006	NADH:ubiquinone reductase (H^+^-translocating)
	EC 2.7.7.13	0.291	0.270	0.004	LE	2.025	0.011	Mannose-1-phosphate guanylyltransferase
	EC 3.2.1.23	0.585	0.549	0.008	LE	2.249	0.020	Beta-galactosidase
	EC 3.2.1.51	0.382	0.352	0.006	LE	2.175	0.013	Alpha-L-fucosidase
	EC 3.4.21.102	0.400	0.377	0.004	LE	2.062	0.003	C-terminal processing peptidase
	EC 5.2.1.8	1.219	1.185	0.008	LE	2.195	0.018	Peptidylprolyl isomerase
	EC 5.4.99.2	0.181	0.156	0.004	LE	2.104	0.001	Methylmalonyl-CoA mutase
	EC 2.2.1.6	0.298	0.324	0.004	HE	2.130	0	Acetolactate synthase
	EC 2.3.1.15	0.094	0.115	0.004	HE	2.037	0.004	Glycerol-3-phosphate 1-O-acyltransferase
	EC 2.7.1.69	0.141	0.181	0.011	HE	2.303	0.009	Protein-N(pi)-phosphohistidine-sugar phosphotransferase
	EC 2.7.13.3	0.477	0.513	0.009	HE	2.247	0.009	Histidine kinase
	EC 3.4.16.4	0.261	0.286	0.003	HE	2.075	0	Serine-type D-Ala-D-Ala carboxypeptidase
	EC 3.6.3.17	0.108	0.128	0.006	HE	2.002	0.011	Monosaccharide-transporting ATPase
Fungal	EC 2.3.1.48	1.149	1.060	0.015	LE	2.646	0.006	Histone acetyltransferase
	EC 2.4.1.16	0.536	0.486	0.013	LE	2.396	0.019	Chitin synthase
	EC 3.2.1.18	1.825	1.736	0.021	LE	2.659	0.010	Exo-alpha-sialidase
	EC 3.2.1.3	2.166	1.922	0.052	LE	3.088	0.024	Glucan 1,4-alpha-glucosidase
	EC 4.6.1.2	0.063	0.042	0.007	LE	2.025	0.032	Guanylate cyclase
	EC 5.2.1.8	1.883	1.756	0.030	LE	2.803	0.012	Peptidylprolyl isomerase
	EC 1.14.13.12	0.077	0.123	0.011	HE	2.381	0.042	Benzoate 4-monooxygenase
	EC 2.3.1.85	0.036	0.060	0.006	HE	2.104	0.035	Fatty-acid synthase
	EC 3.1.1.3	0.509	0.530	0.005	HE	2.035	0.029	Triacylglycerol lipase
	EC 3.2.1.58	0.486	0.539	0.012	HE	2.419	0.003	Glucan 1,3-beta-glucosidase
	EC 3.4.17.21	0.149	0.172	0.005	HE	2.043	0.013	Glutamate carboxypeptidase II
	EC 3.5.1.4	0.271	0.305	0.010	HE	2.244	0.046	Amidase
	EC 4.1.1.1	0.129	0.155	0.004	HE	2.104	0.001	Pyruvate decarboxylase

*LE* Low energy, *HE* High energy

No functional features were differed by energy sources

### Several taxa of bacteria, fungi, and protozoa exhibit correlation to lactation traits

**Fig. 6:**
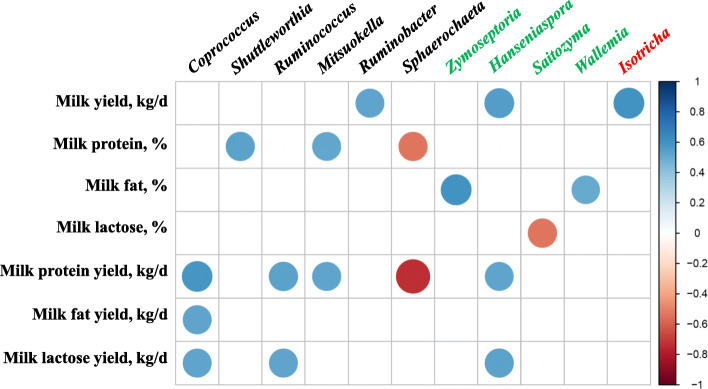
Correlations between major classified genera (each having a relative abundance ≥0.1% in at least one of the dietary treatments; prokaryotes, fungi, and protozoa are labeled in black, green, and red, respectively) and milk quality and lactation performances. Only those with significant and strong correlation coefficients (|*r*| > 0.5, *P* < 0.05) were visualized. The degree of each correlation was shown both with the size and intensity of the color based on the color key on the right side

Significant and strong correlations (|*r*| > 0.5, *P* < 0.05) between some of the lactation traits and several major genera were found (Fig. 6). *Coprococcus*, *Shuttleworthia*, and *Ruminococcus*, all of which were increased by the HE diets, were positively correlated with one or more of the lactation traits, either proportion or yield of major milk components, whereas *Sphaerochaeta*, which was increased in the LE diets, exhibited a negative correlation with both milk protein content and yield. *Mitsuokella*, *Ruminobacter*, *Hanseniaspora* (a fungal genus), which were enriched by or specifically associated with the SFC diets, also showed a positive correlation with several lactation traits. The fungal genus *Saitozyma* showed a negative correlation with milk lactose yield, while the protozoal genus *Isotricha* has a positive correlation with milk yield.

## Discussion

Many nutritional factors affect the quality and quantity of milk produced by dairy cows [28]. Dietary manipulation is most commonly used to maximize lactation performance [2], which includes forage-to-concentrate ratio, grain processing, feed particle size, and forage length (e.g., [29]). Milk production by dairy cows is a highly energy-dependent process [30]. Indeed, in our previous study, the HE diets significantly increased milk protein content and the yield of milk, milk protein, and lactose although they did not affect milk fat or lactose [9]. The yield of milk and milk protein was also increased by SFC in the cows fed the LE diets. It was shown that the HE diets increased crude MP yield and total-tract apparent digestibility, and crude MP yield was also increased by SFC in the cows fed the LE diets. Apparently, lactation performance is strongly associated with rumen microbiota, yet, few studies have focused on the effect of dietary energy level or source on rumen microbial shifts even though many studies have investigated the effect of the forage-to-concentrate ratio. Focusing on how both dietary energy source and level affect the multi-kingdom microbiota in dairy cows that showed different lactation performance [9], we identified taxa of rumen microbes that might be associated with some of the important lactation traits as affected by dietary energy level (1.53 vs. 1.72 Mcal/kg of dry matter) but not energy source (ground vs. steam-flaked corns).

### Effect of dietary energy source and level on the prokaryotic, fungal, and protozoal rumen microbiota

The two different dietary energy levels resulted in differences in the diversity of the rumen prokaryotic microbiota, with the LE diets increasing species and genus richness. The positive correlation between species richness and the fiber content in diets has been demonstrated in several studies on dairy heifers and cows [31–33]. The increase in species richness by the LE diets is consistent with the above studies and corroborates the needs for concerted actions of a diverse microbiota to effectively digest recalcitrant feed fiber. The two energy sources only led to a significant difference in ASV richness and phylogenetic diversity, with the GC increasing both alpha diversity measurements. Although ASVs may not necessarily represent strains of microbes, the similar effects of both the energy source and energy level on species and genus richness, but not on ASV richness, may suggest that the two energy sources might have caused the difference in richness at the subspecies level. Based on our literature search, no study has been reported that examined the effect of ground vs. steam-flaked corns on rumen microbiota. It remains to be determined if the differences in digestibility between ground corn and steam-flaked corn may select different amylolytic bacteria. The two energy levels also shaped the fungal microbiota differently, though to a lesser extent than prokaryotic microbiota. The difference in fiber content between the two energy levels is likely attributable to the observed fungal effect. The relatively small effect of energy level on the fungal microbiota and the lack of impact on the protozoal microbiota are consistent with a previous report that the overall rumen protozoal and fungal microbiotas were slightly more stable than the prokaryotic microbiota in dairy cows fed diets with different forage-to-concentrate ratios [33]. The two energy sources did not cause much difference in the rumen microbiota, prokaryotic, fungal, or protozoal. This may be attributed to the lack of significant difference in digestibility between ground and the steam-flaked corns [9]. Collectively, these results suggest that under the dietary treatments of the present study, the dietary levels had a greater impact on the rumen microbiota than energy source (ground vs. steam-flaked corns).

### Effects of energy source and level on individual taxa of the rumen microbiota

*Ruminobacter* and *Shuttleworthia* were increased by the HE diets, which can be explained by their ability to utilize starch [34, 35]. *Ruminococcus*, *Coprococcus*, *Butyrivibrio*, and *Shuttleworthia*, all of which are within the phylum Firmicutes, were considered as the core genera of rumen bacteria in large dairy cohorts [36]. Their relative abundance was increased by the HE diets. *Ruminococcus* and *Butyrivibrio* are generally considered as fibrolytic bacteria in the rumen, but some of their species also are amylolytic [37, 38], such as *R. bromii* and *B. fibrisolvens*, both of which could increase their growth in response to starch-rich diets [39]. However, those two bacterial genera were more frequently prevalent in cows fed forage-based diets [40]. Therefore, absolute quantification of several representative species of those major culturable bacteria is required to determine their population shifts under different dietary treatments. Three uncultured/unidentified candidate genera within the phylum Bacteroidetes, including CF231, YRC22, and BF311, were increased by the LE diets. Those three genera were significantly more predominant in the rumen of hay-fed goats compared to high-grain fed goats [41] and high-producing dairy cows (except for BF311) [42]. Another candidate genus (i.e., RFN20) within Firmicutes was also increased by the LE diets. These candidate genera were repeatedly found as major taxa, but the lack of cultured representatives and information on their metabolism hinders the interpretation of their shifts under given dietary treatments. The increased relative abundance of *Sphaerochaeta* and *Anaeroplasma*, both of which can use starch but not fiber, was consistent with the report on sheep fed corn stover [43] and on Holstein heifers fed a diet with increasing forage-to-concentrate ratio [33]. *Moryella*, which utilizes glucose, galactose, maltose, and ribose, but not starch [44], was previously linked to milk fatty acid profiles [45] and milk fat content [36]. In our previous study, however, milk fatty acid profiles were not determined and the milk fat was not affected by either energy source or level [9]. Other factors besides readily fermentable energy sources or levels might probably also shift its population. The increased relative abundances of two genera within Firmicutes, *Mitsuokella*, and *Selenomonas*, observed in the cows fed the SFC diets might be partially explained by increased digestibility of the steam-flaked corn compared to the ground corn. The readily fermentable SFC might have provided better niches for the starch-degrading bacteria resulting in increased lactation traits including the yield of fat-corrected milk (FCM), energy-corrected milk (ECM), milk protein, lactose, and content of milk protein and non-fat solid compared to the ground corn [9].

*Saccharomyces* was the only fungal genus having a higher relative abundance in the cows fed the HE diets compared to the LE diets. The increase of yeast is consistent with its occurrence in silage and the rumen of grain-fed cows [46]. The LE diets increased the predominance of eight genera of fungi, but interestingly, none of them is members of the phylum Neocallimastigomycota, to which all the six recognized and candidate genera of rumen fungi belong [47]. Based on our literature search, only *Cryptococcus* has been detected in the rumen of goats [48] and musk oxen [49]. Few studies have targetedly analyzed the fungi in ruminant nutritional studies. Future research can further determine their occurrence and potential function in the rumen. The two energy sources only affected two genera of fungi, with *Hanseniaspora* being enriched by the SFC diets and *Aspergillus* being enriched by the GC diets. As a genus of yeast related to *Saccharomyces*, *Hanseniaspora* might have been increased due to the increased digestibility of SFC. The ability to use different carbohydrates including cellulose [50] might explain the higher predominance of *Aspergillus* resulted from the GC diets compared to the SFC diets. The higher relative abundance of *Methanobrevibacter* in the rumen of the GC-fed cows could enhance the fungal activity to break down the plant materials, as suggested in another study [51]. However, the differences of fungal profiles among the dietary treatments were possibly caused by the transient contamination from the forage intake which has been reported in a previous study [52]. Thus, the metabolic contribution of some of the fungi observed in the rumen might be limited and need to be interpreted with caution.

The differential relative abundance of *Polyplastron* and *Entodinium* in response to the LE and the HE diets, respectively, was possibly due to the different levels of fiber contents in the diets as previously reported in Holstein heifers [33]. *Polyplastron* is one of the largest entodiniomorphs which can engulf and digest fibrous feed particles inside its cells using its cellulases [53]. The dominance of the amylolytic *Entodinium* spp. has been repeatedly observed in dairy cattle fed high-grain diets [33, 54]. However, *Dasytricha* is considered a saccharolytic protozoa genus with no known cellulolytic ability [55]. Besides the present study, only one study reported an increase in *Dasytricha* predominance by high forage diets [56]. Using refaunated dairy goats as a model, one study showed a negative correlation between *Dasytricha* and MP synthesis [57]. The higher predominance of this protozoal genus and the lower lactation performance of the cows fed the LE diets [9] corroborate the aforementioned study [57]. Future research is warranted to further investigate how diets affect *Dasytricha* and its relationship with MP synthesis in the rumen.

### Functional features shaped by different dietary energy levels and sources

As expected from the differential prokaryotic microbiota diversity and composition, dietary energy levels significantly affected the overall predicted functional features. Besides, a greater number of functional features were predicted in the rumen microbiota of the cows fed the LE diets compared to the HE diets, irrespective of the reference databases used. On the other hand, the two energy sources (ground vs. steam-flaked corns) did not cause any significant difference in the predicted prokaryotic functions. This may be attributable to the lack of sensitivity or resolution (the data were based on relative abundance) the analysis methods or the high functional redundancy in the rumen microbiota [58]. However, the LE diets significantly increased the number of functional features compared to the HE diets. Furthermore, LEfSe analysis showed differentially abundant enzymes only between the two different dietary energy levels, and some of them are enzymes involved in carbohydrate metabolisms (e.g., 2-oxoacid oxidoreductase, 2-oxoglutarate synthase, succinate dehydrogenase, protein-N(pi)-phosphohistidine-sugar phosphotransferase, monosaccharide-transporting ATPase, glucan 1,4-alpha-glucosidase, pyruvate decarboxylase, and glucan 1,3-beta-glucosidase). The increased recalcitrance of the LE diets has probably created more niches and the requirement for more complex metabolic functions to digest the increased forage components. The recently updated fungal reference genome database in PICRUSt2 allows the prediction of fungal functions from ITS1 sequence data [24]. Unfortunately, the functional prediction from rumen protozoa is still not possible because of the lack of genomic information for any of the rumen protozoa.

### Microbial taxa potentially driving the difference in the predicted functional pathways between the two dietary energy levels

To gain insights into the microbial underpinning of nutritional of functional changes, the microbes to which the observed changes can be attributable need to be identified. However, it is still difficult with complex high-dimensional microbiota data. FishTaco is an analytical and computational framework that integrates comparative taxonomic and functional analysis results to accurately quantify taxa contributing to disease-associated functional shifts [25]. It has been used in many studies on human microbiota, but only in one study on the rumen microbiota [59]. In the present study, CF231, a candidate genus-level taxon of the family Paraprevotellaceae, was identified to be the potential taxon contributing to the synthesis of primary and secondary bile acids in the rumen of the cows fed the LE diets. The prevalence of CF231 in the rumen was reported previously [36], and its abundance was negatively correlated to milk yield [42] but positively correlated with milk protein content [36]. Enriched secondary bile acid biosynthesis was found in beef cattle with high residual feed intake in a metatranscriptomic study of rumen microbiota [60]. Given the limited amount of bile acids present in the rumen, the inferred relationship between bile acid metabolism in the rumen and feed efficiency or milk production of dairy cows is intriguing. The unknown metabolism of CF231 adds more mist to this mystery.

Enriched ABC transporters and pyruvate metabolism in response to the HE diets possibly related to the increased sugar transport and VFA production, respectively [61]. The abundance of those pathways was also shown to vary depending on the dietary treatment [62], methane yield [63], and feed efficiency [64]. Furthermore, biosynthesis pathways for seven amino acids and glycerophospholipid metabolism were also enriched by the HE diets. The increased gene abundance of amino acid biosynthesis can be explained as an increase in the growth of the taxonomic drivers by the HE diets and is consistent with the increase of lactation performance in the cows fed the HE diet [9]. *Ruminococcus* was the contributor that explains several HE-enriched pathways: biosynthesis of amino acids (valine, leucine, isoleucine, lysine, phenylalanine, and tyrosine, tryptophan) and metabolism of glycerophospholipid, pyruvate, nicotinate, and nicotinamide. Both *Ruminococcus* and *Coprococcus* contributed to the enrichment of ABC transporters. When we searched the IMG database [65] that contains 29 sequenced genome of *Ruminococcus* isolates of rumen origin and did the KEGG annotation using the KEGG Automatic Annotation Server [66], we found all the HE-enriched KEGG pathways (data not shown). *Coprococcus*, which uses the acrylate pathway for propionate production from lactate, was more predominant in efficient dairy cows [5]. Also, Lachnospiraceae, which harbors several genera of ruminal bacteria including *Coprococcus*, *Butyrivibrio*, and *Shuttleworthia*, was previously shown to be positively correlated with milk production and milk protein content, though not with milk fat content [67]. It has been consistently recognized that the Firmicutes-to-Bacteroidetes ratio was positively linked to feed efficiency, milk production, and milk fat yield [4]. The increase of Lachnospiraceae abundance corresponded to a significantly higher (*P* = 0.0005) Firmicutes-to-Bacteroidetes ratio (data not shown) in the HE diet group, and this might be partially associated with the efficient conversion of the HE diets to milk observed in the previous study [9].

### Effect of energy source and level on microbial interactions

The two dietary energy sources and levels resulted in some differences in the co-occurrence networks, with considerably more fungal nodes being found for the LE and the GC diets. Each of the four diets also resulted in a specific co-occurrence network. Although the co-occurrence networks were based on correlation in relative abundance, some of them may reflect biological interactions. However, it is not feasible to verify or test if a co-occurrence relationship is the consequence of biological interactions. Among the major fungal microbiota, the cellulolytic genera *Acremonium*, *Cladosporium*, and *Penicillium* [68, 69] were exclusively found in the co-occurrence networks corresponding to the LE or the GC diets, but those fungi are not commonly found in the rumen. Their actual residence and contribution need further verification. Among the known common ruminal fungi, *Orpinomyces* was exclusively found in the rumen of the cows fed the LE diets. This is consistent with the prevalence of this fungal genus previously found in the rumen of cattle fed a high-fiber diet [70]. Even though some of the fungal taxa detected in the present study might be transient, their exclusive interactions with many of the other microbial taxa exclusively for a specific dietary treatment is interesting. It should be noted that even though the numbers of specific nodes of prokaryotes were smaller than those of fungi or protozoa, the total numbers of nodes of prokaryotes were much larger for any of the diets. Thus, ruminal prokaryotes had more extensive interactions than rumen fungi or protozoa. Future research is needed to adequately interpret co-occurrence in the context of lactation performance.

### Correlations between microbial genera and lactation traits

Among the 11 microbial genera that showed a significant and strong correlation with at least one of the lactation traits determined, *Ruminobacter*, *Hanseniaspora*, and *Isotricha* might be positively associated with milk yield, while *Coprococcus*, *Ruminococcus*, *Mitsuokella*, and *Hanseniaspora* might be associated with milk protein yield. *Sphaerochaeta* and *Saitozyma* exhibited negative correlations with two (milk protein content and milk protein yield) and one (milk lactose content) lactation traits, respectively. Little is known about the function of these genera in the rumen. Because correlation does not imply causation, future research is needed to determine their relationship with milk production. Some of these genera, however, may be used as potential biomarkers of lactation traits.

## Conclusions

Dietary energy levels markedly affected the multi-kingdom microbiota and microbial functions, whereas the energy sources (ground corn vs. steam-flaked corn) had little effect. Some microbial taxa and metabolic pathways were enriched by the high energy level, and they might contribute to the improved lactation performance observed previously. Furthermore, because some of the microbial genera enriched by the high energy diets had exhibited increased predominance in high producing cows, they may be used as biomarkers of one or more of the lactation traits. This study also showed that steam-flaked corn also benefited several microbial taxa, which are associated with high lactation performance. Several uncommon fungi were also affected, especially by the energy level. Specific co-occurrence among different taxa resulted from both the energy level and source (particularly energy level). Future research is warranted to further investigate if the taxonomic drivers identified with FishTaco are the cause of the differential metabolic pathways and how the co-occurrence network can be interpreted in the context of rumen fermentation characteristics and lactation performance.

## Supplementary information

**Additional file 1: Figure S1.** Relative abundance of the major bacterial and archaeal phyla (A) and genera (B), fungal phyla (C) and genera (D), and protozoal genera (E) (one those each having a relative abundance ≥0.1% in at least one of the dietary treatments are shown). **Table S1.** Feed and nutrient composition of the diets. **Table S2.** Primers used for amplicon sequencing.

## Data Availability

The raw phylogenetic marker gene sequences were deposited in the NCBI SRA under BioProject PRJNA523854. All the analyzed microbial datasets in the current study are available from the corresponding author on reasonable request.

## Abbreviations

SARA: Subacute rumen acidosis
CS: Corn stover
ME: Metabolizable energy
MP: Microbial protein
SFC: Steam-flaked corn
GC: Ground corn
LE: Low energy
HE: High energy
SRA: Sequence Read Archive
ITS: Internally transcribed spacer
ASV: Amplicon sequence variant
PCoA: Principal coordinates analysis
SCNIC: Sparse Co-occurrence Network Investigation for Compositional data
CoDiNA: Co-expression Differential Network Analysis
EC: Enzyme Commissions
KEGG: Kyoto Encyclopedia of Genes and Genomes
PCA: Principal components analysis
FishTaco: Functional Shifts’ Taxonomic Contributors
PICRUSt2: Phylogenetic Investigation of Communities by Reconstruction of Unobserved States 2
PERMANOVA: Permutational multivariate analysis of variance
LDA: Linear discriminant analysis
LEfSe: Linear discriminant analysis effect size
NDF: Neutral detergent fiber
FCM: Fat-corrected milk
ECM: Energy-corrected milk
VFA: Volatile fatty acid
IMG: Integrated microbial genomes
