# Supplementary material for: Dietary energy sources and levels shift the multi-kingdom microbiota and functions in the rumen of lactating dairy cows
Source: J Anim Sci Biotechnol. 2020 Jun 22;11:66. doi: 10.1186/s40104-020-00461-2 (PMC7310258; doi:10.1186/s40104-020-00461-2)
Supplement: Supplementary file 1 — Additional file 1: Figure S1. Relative abundance of the major bacterial and archaeal phyla (A) and genera (B), fungal phyla (C) and genera (D), and protozoal genera (E) (one those each having a relative abundance ≥0.1% in at least one of the dietary treatments are shown). Table S1. Feed and nutrient composition of the diets. Table S2. Primers used for amplicon sequencing. [file 40104_2020_461_MOESM1_ESM.pptx]

## Slide 1
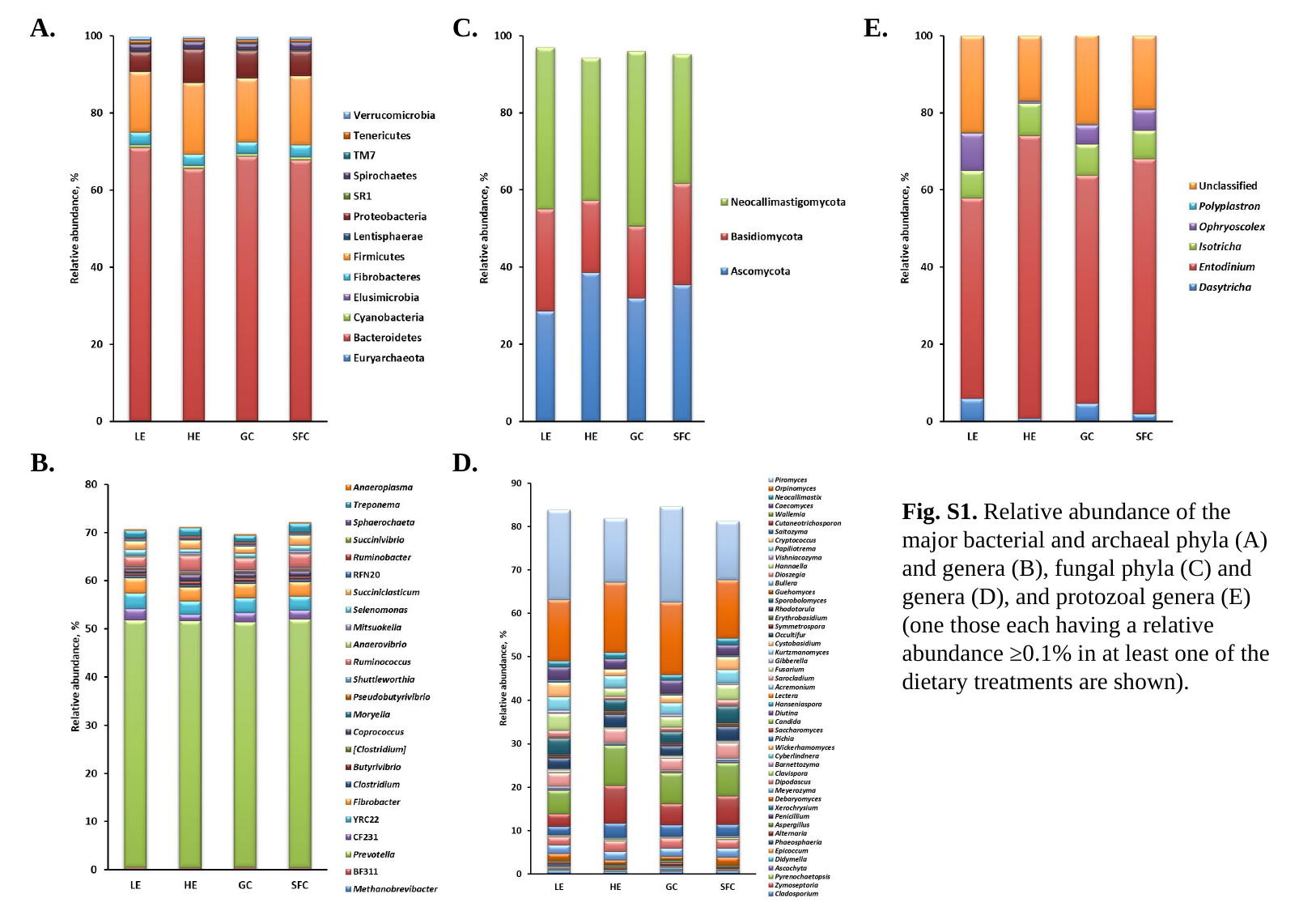

A.
C.
E.
B.
D.
Fig. S1. Relative abundance of the major bacterial and archaeal phyla (A) and genera (B), fungal phyla (C) and genera (D), and protozoal genera (E) (one those each having a relative abundance ≥0.1% in at least one of the dietary treatments are shown).
